# Race Improvement

**Published:** 1914-02-28

**Authors:** Binnie Dunlop

**Affiliations:** Alexandra Court, S.W.


*To the Editor of* The Hospital.

### Race Improvement.

*To the Editor of* The Hospital.

Sir,—I feel grateful to you for saying about eugenics that "the less we hurry into drastic measures the better." What is urgently needed for race improvement, and for most of our other social problems, is the Neo-Malthusian doctrine of early marriage and small families. This is now bound to come, and all thoughtful people should study it.

In the meantime, doctors and nurses must realise that it is dysgenic, as well as cruel, to leave parents ignorant of family limitation when they have as many children as their means or health justify.—I am, Sir, etc.,

Binnie Dunlop, M.B., Ch.B.

Alexandra Court, S.W.

February 21, 1914.

